# Supplementary material for: The interactome of intact mitochondria by cross-linking mass spectrometry provides evidence for coexisting respiratory supercomplexes
Source: Mol Cell Proteomics. 2017 Dec 8;17(2):216–32. doi: 10.1074/mcp.RA117.000470 (PMC5795388; doi:10.1074/mcp.RA117.000470)
Supplement: Supplemental Data [file supp_RA117.000470_133922_0_supp_23978_fzffwf.zip › spectra_annotation/mito_spectra_annotation/447-1-51-1-11-1.pdf]

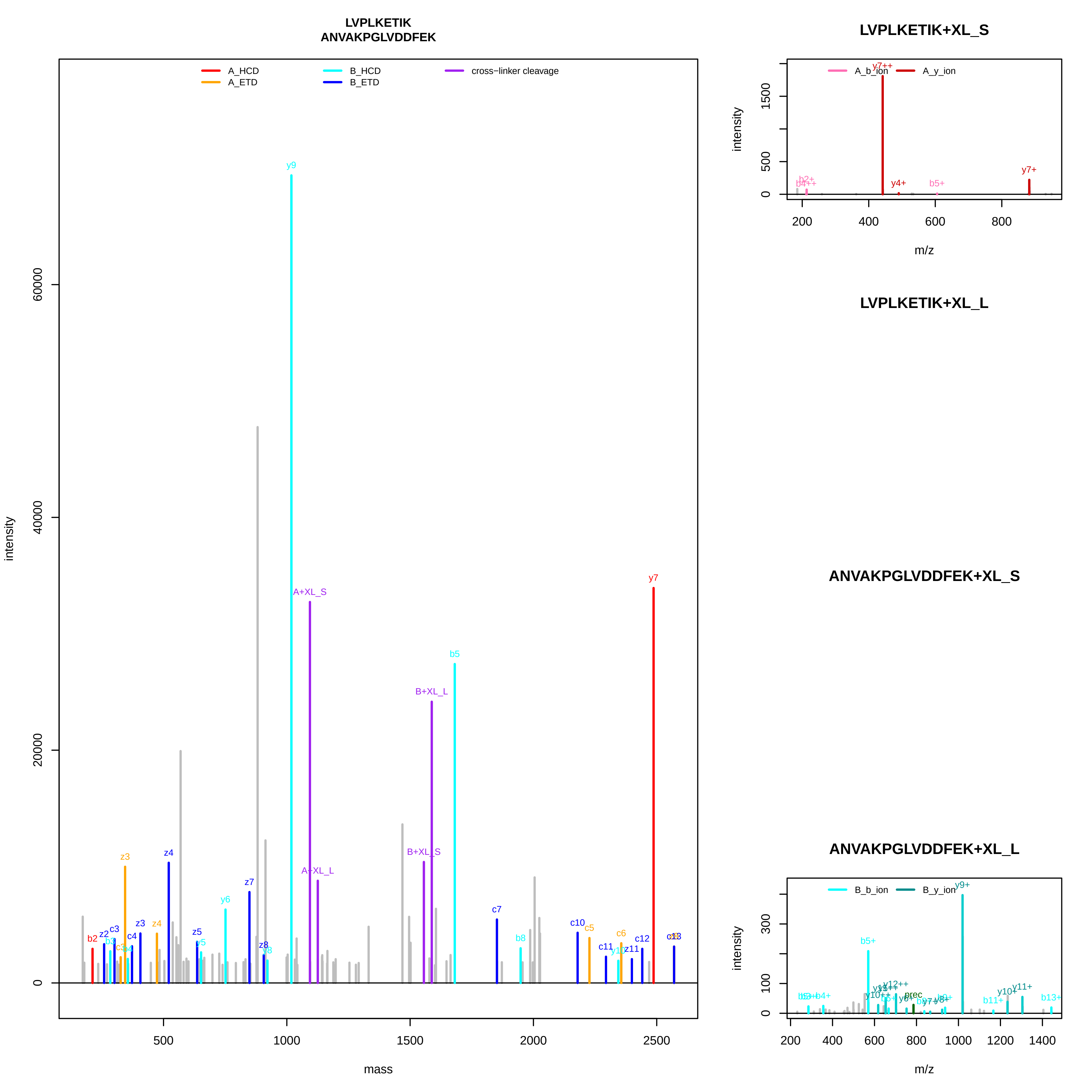
LVPLKETIK
ANVAKPGLVDDFEK
A_HCD
A_ETD
B_HCD
B_ETD
cross−linker cleavage
intensity
mass
0
20000
40000
60000
500
1000
1500
2000
2500
y9
A+XL_S
A+XL_L
B+XL_L
B+XL_S
b5
y7
z3
z4
y6
z7
z5
y5
z8
y8
c7
b8
c10
c5
c6
c11
y11
z11
c12
c13
b2
z2
c3
b3
c4
z3
LVPLKETIK+XL_S
A_b_ion
A_y_ion
y7++
y7+
y4+
b5+
b2+
b4++
0
500
1500
200
400
600
800
m/z
LVPLKETIK+XL_L
ANVAKPGLVDDFEK+XL_S
ANVAKPGLVDDFEK+XL_L
B_b_ion
B_y_ion
y9+
b5+
y12++
y10+
y11+
b13+
b11+
prec
y8+
y7+
y6+
b4+
0
100
300
200
400
600
800
1000
1200
1400
